# Preliminary Psychometric Evaluation of the Arabic Version of the NURS-DIAL Questionnaire Among Nursing Students in Saudi Arabia: Cross-Sectional Study

**DOI:** 10.2196/106044

**Published:** 2026-10-30

**Authors:** Nader Alnomasy, Habib Alrashedi, Petelyne Pangket

**Affiliations:** 1 Medical Surgical Nursing Department, University of Ha'il, PO Box 2240, Ha'il, 55349, Saudi Arabia, 966 532476596; 2 Community Nursing Department, Taif University, Taif, Mecca Region, Saudi Arabia

**Keywords:** digital literacy, mHealth literacy, artificial intelligence literacy, nursing students, digital well-being

## Abstract

**Background:**

The rapid progress in digital transformation and the increasing reliance on AI in health care have created an urgent need for validated assessments of digital and AI literacy among nursing students. However, there is a substantial gap in culturally adapted assessment tools for Arabic-speaking populations.

**Objective:**

This study aimed to translate, culturally adapt, and conduct a preliminary psychometric evaluation of the Arabic version of the Nursing Digital and AI Literacy Assessment (NURS-DIAL).

**Methods:**

This cross-sectional study evaluated the Arabic-translated and culturally adapted version of the NURS-DIAL. The psychometric evaluation included content validity, confirmatory factor analysis (CFA) using diagonally weighted least squares estimation, internal consistency reliability, construct validity, and known-groups validity. Given the complexity of the 52-item, 5-factor NURS-DIAL model and the limited sample size, the CFA findings should be interpreted as preliminary.

**Results:**

Content validity was strong, with item-level content validity indices ranging from 0.83 to 1.00 and a scale-level content validity index average of 0.94. CFA provided preliminary support for a 52-item, 5-factor model after removing 4 items. Model fit indices were as follows: comparative fit index was 0.930, Tucker-Lewis index was 0.926, root mean square error of approximation was 0.055, standardized root mean square residual was 0.103, and goodness-of-fit index was 0.947. The internal consistency estimates ranged from 0.856 to 0.919. Selected known-group comparisons also provided preliminary validity evidence.

**Conclusions:**

The NURS-DIAL demonstrated preliminary evidence of content validity, structural validity, internal consistency reliability, and construct validity among nursing students at 1 university in Saudi Arabia. Because of the limited sample size and single-institution setting, the tool should currently be used for exploratory research and formative assessment in similar settings. Larger multi-institutional studies are needed to confirm its factor structure and broader applicability.

## Introduction

The rise of digitalization in health care and nursing education has created an increasing demand to evaluate nursing students’ competence in using digital and AI technologies. Contemporary digital competency models in higher education emphasize 5 key domains: information literacy, digital well-being, digital research, content creation, and online identity management as essential for safe and effective technology use in learning and practice [1]. Recent studies have identified gaps in nursing students’ self-perceived competence in information literacy, digital research, and digital innovation, while also highlighting the importance of digital health literacy, digital skills, and positive attitudes toward AI [2,3]. However, studies in the Gulf and wider Arab region have documented gaps among nursing and health professions students in information handling, critical appraisal, and digital self-management [4-6]. Moreover, a recent study of e-learning adoption in Gulf Cooperation Council (GCC) higher education institutions showed that students’ use of e-learning systems was influenced by their perceptions of system usability and accessibility. These perceptions may affect students’ ability to engage effectively with online learning environments [7].

The role of AI in nursing practice has grown rapidly in recent years. Nurses now use AI in clinical decision-support systems, diagnostic imaging, predictive analytics, documentation, patient monitoring, and robotic technology. As AI becomes more integrated into patient care, nurses need to understand how to use it safely. This includes foundational knowledge of basic AI principles, the ability to use AI-supported tools appropriately, and the use of clinical judgment when considering AI-generated recommendations [8,9]. Therefore, nursing students should receive exposure to and training in AI, including their roles and responsibilities, before entering clinical practice [10,11].

In Saudi Arabia, nursing students displayed a wide range of perceptions and readiness for AI [12]. Some students expressed a lack of knowledge of and uncertainty about the concept of AI, though a majority were positively inclined to the technology [12]. Students with higher AI literacy expressed a greater sense of empowerment and a reduction in their fears about the technology [13]. Systematic reviews of the nursing literature on AI indicated that the integration of AI education programs in nursing programs can reduce nursing students’ fears and improve their understanding of the technology [14]. A review of the literature on the concept of AI in the Middle East and North Africa indicated that a majority of the studies conducted and published on the topic originated from that region, but the majority of the studies did not use instruments developed within those countries to measure students’ attitudes and readiness to use the technology [9].

There is a significant amount of nursing literature that discusses how Middle Eastern and North African countries feel about AI in nursing education. Some of the areas that have been studied within this region include nurses’ attitudes toward AI, their literacy regarding the technology, their readiness for the technology, and their intentions to adopt AI into nursing education [9]. Despite the wealth of studies conducted within this region, there is limited reporting in the literature of how these studies were performed. In many of the Arabic language nursing education texts, the scales and questions used in the studies were developed in non-Arabic speaking countries, and there has been limited reporting of how these scales have been formally translated, culturally adapted, and psychometrically evaluated for use within the Middle Eastern and North African countries. There is a need not only to describe nursing students’ attitudes and readiness for AI but, more importantly, to establish culturally appropriate measures for assessing digital and AI-related skills in Arabic-speaking educational environments. This study addressed this need by conducting an Arabic adaptation and preliminary psychometric analysis of the Nursing Digital and AI Literacy Assessment (NURS-DIAL).

The NURS-DIAL was developed to assess 5 related domains among nursing students: information literacy, mobile health (mHealth) literacy, AI literacy, digital well-being, and perceived AI education topics. The present study aimed to translate, culturally adapt, and conduct a preliminary psychometric evaluation of the Arabic NURS-DIAL among nursing students in Saudi Arabia. The study examined content validity, internal consistency, structural validity using confirmatory factor analysis (CFA), intersubscale associations, and selected known-group comparisons. Translation and cultural adaptation were undertaken using the stages recommended by Beaton et al [15], including forward translation, synthesis, back-translation, expert-committee review, and pretesting with members of the target population.

## Methods

### Study Design

This methodological cross-sectional study aimed to translate and conduct a preliminary psychometric evaluation of the Arabic version of the NURS-DIAL questionnaire among nursing students.

### Population/Setting/Sampling

Convenience sampling was used to collect data from enrolled nursing students. Eligible students were invited to complete an online survey via institutional announcements and online learning platforms. The sample included undergraduate and postgraduate nursing students of all years of study and specializations in the field. The inclusion criteria were age 18 years or older, ability to read Arabic written language, and electronic informed consent. Exclusion criteria were students enrolled in nonnursing programs and students who participated in the pilot phase of NURS-DIAL. The final sample included 196 nursing students.

### Sample-Size Consideration

The final sample included 196 nursing students. Although this sample exceeded the absolute minimum of 100 participants, it was still small in relation to the complexity of the model. The final instrument contained 52 items across 5 dimensions. Therefore, the participant-to-item ratio was approximately 3.8:1. This ratio is less than the commonly recommended ratios of 5 to 10 participants per item and more conservative recommendations for complex CFA models. In other words, the sample size was adequate for the initial psychometric evaluation but not for the final confirmation of the factor structure. The CFA findings should be viewed as preliminary and verified in further studies using larger independent samples. Additionally, future studies should use a priori model-based sample size estimation or Monte Carlo power analysis that considers the number of factors and indicators, expected factor loadings, residual variances, correlations between factors, ordinal response categories, estimator type, and anticipated missing values.

### Development and Governance of the NURS-DIAL

The NURS-DIAL was created through a multinational research collaboration (AI4Health consortium) to evaluate nursing students’ knowledge of digital and AI literacy in 5 areas. Four previously used tools were integrated into the NURS-DIAL: the information literacy and digital well-being subscales of Martzoukou et al [2], Discipline-Based Digital Competence Survey Battery, mHealth Literacy Scale of Kim et al [16], Artificial Intelligence Literacy Scale of Wang et al [17], and Education Topics Needs Survey of Civaner et al [18] on AI.

The initiative involves nursing faculties and departments in several countries: UMIT TIROL in Austria, the University of Maribor Faculty of Health Sciences in Slovenia, and the University of Hail in Saudi Arabia, where the current sample was recruited. All participating institutions follow a common research protocol, obtain approval from their local ethics committees, and translate and culturally adapt the questionnaire for their own settings.

The consortium coordinating team manages the governance of the instrument, including curation of the item pool and cross-site coordination, while the project coordinators manage requests to use the instrument through the NURS-DIAL project website. Researchers and collaborating institutions can use the questionnaire at no cost. In the present study, the questionnaire was administered locally using Qualtrics and Google Forms. Parallel validation work is underway in additional cohorts, and preliminary findings have been reported in an Austrian cohort. To our knowledge, this study is the first to present an Arabic-language adaptation and preliminary psychometric evaluation of the NURS-DIAL.

The NURS-DIAL research team added 4 items to the AI Literacy subscale to better reflect AI literacy in the context of nursing and health care, as the original instrument was developed for general users, expanding the original 12-item scale [17] that measured AI literacy. Also, the AI Education Topics subscale was expanded from 15 to 20 items to include additional AI-related curricular priorities determined to be relevant to nursing education.

The Information Literacy subscale consists of 8 items, using a 5-point rating scale from novice to expert. The mHealth Literacy subscale consists of 6 items, using a 5-point rating scale from strongly disagree to strongly agree. The AI Literacy subscale consists of 16 items, using a 7-point rating scale from strongly disagree to strongly agree. The Digital Well-being subscale consists of 6 items, using a 5-point rating scale from novice to expert. The AI Education Topics subscale includes 20 items on a 5-point scale from include to no need describing the perceived priorities for AI content in the nursing curricula by students. Four items (AIL_c, AIL_g, DW_e, and AIE_t) were deleted after CFA, resulting in a final Arabic NURS-DIAL of 52 items.

### Translation, Cultural Adaptation, Content Validity, and Pretesting

The NURS-DIAL was translated and culturally adapted into Arabic using the 5-stage framework described by Beaton et al [15] for cross-cultural adaptation of self-report measures. Permission to translate, adapt, and publish the questionnaire was obtained from the AI4Health consortium, and copyright requirements were met.

In stage 1, 2 bilingual translators independently translated the original English NURS-DIAL into Arabic. Both translators were native Arabic speakers, fluent in English, and had backgrounds in nursing or health sciences. In stage 2, the interdisciplinary research team reviewed and reconciled the 2 translations. The review focused on terms related to digital technology, AI, and reverse-worded items to ensure clarity and cultural relevance in the Saudi Arabic version.

In stage 3, the reconciled Arabic version was independently back translated into English by 2 bilingual translators whose first language was English, who were fluent in Arabic, and who were unfamiliar with the original questionnaire. In stage 4, a multidisciplinary expert committee reviewed the forward translations, reconciled Arabic version, and back-translations. The committee assessed semantic, idiomatic, experiential, and conceptual equivalence and produced a prefinal Arabic version of the NURS-DIAL.

Six experts reviewed the prefinal 56-item Arabic NURS-DIAL for content validity. The panel included 3 nursing education academics with 8, 12, and 15 years of experience, a health informatics expert with 10 years of experience, a linguist with 7 years of experience, and a psychometrician with 11 years of experience. All held doctoral degrees and had experience in instrument development. Experts rated each item for relevance, clarity, and cultural appropriateness on a scale from 1 (not relevant) to 4 (highly relevant). The item-level content validity index (I-CVI) values for all items were at least 0.83, and the scale-level content validity index average was 0.94. Based on the expert review, minor wording changes were made to 4 items to improve clarity and cultural appropriateness for use in Saudi Arabia.

In stage 5, the prefinal Arabic NURS-DIAL was tested with 35 nursing students who met the same eligibility criteria as the main study sample. After completing the questionnaire, students commented on the clarity of the items, wording, response options, time needed to complete the questionnaire, and cultural relevance. It took approximately 15 to 20 minutes to complete. Students did not report any concerns about cultural appropriateness, and no items were added. The research team reviewed the feedback before the main psychometric evaluation. The Arabic items, English backtranslations, response options, scoring directions, and item-retention status are provided in Table S4 in Multimedia Appendix 1.

### Data Collection Procedure

Participants completed a self-administered online survey using Qualtrics and Google Forms. The survey could be accessed at participants’ convenience using a smartphone, tablet, or computer. The survey link was sent out to nursing students via email, learning management system (LMS), and faculty posts for all program levels and years of study.

All invitation communications to standardize survey administration provided information on the study purpose, eligibility criteria, voluntary participation, and anticipated completion time and directed students to 1 online interface. The first page of the survey contained an information sheet and an electronic consent form, followed by items presented in a fixed sequence. The majority of closed-ended items were required; skip patterns were used only when conceptually justified; and the survey was designed for mobile accessibility to minimize missing data.

### Statistical Analysis

Statistical analysis of the psychometric properties of the Arabic version of the NURS-DIAL was carried out on the main validation dataset. After screening the data, special codes were treated as missing values, and some items were reverse scored. Missingness was patterned, increasing monotonically with item position consistent with survey dropout (Spearman ρ=0.995; Tables S1 and S2 in Multimedia Appendix 1); the primary CFA used weighted least squares mean and variance (WLSMV) with pairwise-present information, and the findings were robust to complete-case and multiple-imputation sensitivity analyses (Table S3 in Multimedia Appendix 1). Structural validity was evaluated with CFA using an appropriate estimator for ordinal data and standard fit indices. Internal consistency was evaluated by Cronbach α (≥0.70 was considered acceptable). Construct validity was assessed by intersubscale correlations for convergent and discriminant validity, while known-group validity was assessed by 2-tailed *t* tests and 1-way ANOVA on subscale scores.

### Sex Reporting

In this study, sex was self-reported by participants in the online survey (male/female) and is used in the known-group validity analyses. Biological sex as recorded in official records was not assessed.

### Ethical Considerations

Prior to beginning data collection, ethical approval was obtained from University of Hail’s Institutional Review Board (case number H-2025‐784). As part of obtaining informed consent, each participant was required to complete an electronic form that included a brief description of the study’s purpose and procedures, potential risks/benefits of participating, as well as a statement indicating that participation was optional and that the student could withdraw or refuse to participate at any point during the completion of the survey and would incur no negative consequences. The survey was completed anonymously and did not include any identifying information.

## Results

Table 1 summarizes the demographic and educational characteristics of the participants. Age data were available for 193 participants, with a mean age of 22.5 (SD 5.9, range 18‐53) years. Among the 196 participants, 118 (60.2%) were female. Most participants were undergraduate BSc nursing students (n=185, 94.4%) and were enrolled in the general nursing program (n=163, 83.2%). Third-year students represented the largest academic-year group (n=60, 30.6%).

**Table 1.** Demographic characteristics of the nursing student sample (N=196).

Characteristic and category	Values
Age (y; n=193), mean (SD; range)	22.5 (5.9; 18‐53)
Sex, n (%)	
Male	77 (39.3)
Female	118 (60.2)
Missing	1 (0.5)
Level of study, n (%)	
Undergraduate (BSc)	185 (98.9)
Graduate nondoctoral (MSc)	11 (1.1)
Year of study, n (%)	
1st year	21 (10.7)
2nd year	46 (23.5)
3rd year	60 (30.6)
4th year	49 (25)
5th year	20 (10.2)
Specialization, n (%)	
General nursing	163 (83.2)
Midwifery	27 (13.8)
Mental health	2 (1.0)
Other	4 (2.0)

Missing responses across questionnaire items ranged from 20.4% to 43.4%, with an overall missingness rate of 31.5%. The proportion of missing responses increased consistently as participants progressed through the questionnaire (Spearman ρ=0.995; *P*<.001), suggesting that some participants may not have completed the survey. Overall, 106 (54.1%) participants completed all 52 questionnaire items. Sensitivity analyses using complete cases and multiple imputation produced similar results; therefore, missing data were not considered to have substantially affected the study findings (Tables S1-S3 in Multimedia Appendix 1).

The final version of the Arabic NURS-DIAL is presented in Table 2. It includes the 5 retained subscales of the NURS-DIAL instrument: mHealth literacy, information literacy, AI literacy, digital well-being, and AI education topics. Each subscale was derived from an existing instrument, with some items modified or eliminated. In total, the final Arabic NURS-DIAL comprised 52 items across the 5 subscales.

All 8 information literacy items were retained for the information literacy scale. Participants responded on a 5-point scale ranging from novice to expert. All 6 mHealth literacy items were retained for the mHealth literacy scale. The responses to this scale were reversed, with higher scores indicating more mHealth literacy. The AI literacy scale was reduced from 16 to 14 items and participants responded on a 7-point scale from strongly disagree to strongly agree. The digital well-being scale was reduced from 6 to 5 items and retained its 5-point novice to expert response scale. Finally, the AI education topics scale was reduced from 20 to 19 items and used a reversed 5-point scale, with higher scores indicating greater importance of these topics.

**Table 2.** Final Arabic Nursing Digital and AI Literacy Assessment (NURS-DIAL) structure after confirmatory factor analysis (CFA): subscales, item counts, response scales, and items removed.

Subscale	Source	Original items	Final items	Items removed	Response scale
Information Literacy	Martzoukou et al [2] 2023	8	8	None	1‐5 (Novice → Expert)
mHealth Literacy	Kim et al [16] 2024	6	6	None	1‐5 (reversed; higher=greater mHealth literacy)
AI Literacy	Wang et al [17] 2023 (expanded)	16	14	AIL_c, AIL_g	1‐7 (Strongly Disagree → Strongly Agree)
Digital Wellbeing	Martzoukou et al [2] 2023	6	5	DW_e	1‐5 (Novice → Expert)
AI Education Topics	Civaner et al [18] 2022 (expanded)	20	19	AIE_t	1‐5 (reversed; higher=greater perceived importance)

The CFA fit indices for the 5-factor Arabic NURS-DIAL model are presented in Table 3. The CFA results provided preliminary support for the proposed 5-factor structure. The model demonstrated acceptable fit according to several indices; however, the standardized root mean square residual (SRMR) exceeded conventional thresholds, and the relatively small sample size in relation to the 52-item model limits confidence in the stability and generalizability of the estimated parameters. The chi-square statistic was significant (*χ*²_1264_=1641.69; *P*<.001), with a *χ*²/*df* ratio of 1.30.

**Table 3.** Confirmatory factor analysis (CFA) fit indices for the 5-factor Arabic Nursing Digital and AI Literacy Assessment (NURS-DIAL) model.

Index	Value
*χ*²_1264_	1641.69 (*P*<.001)
*χ*²/*df*	1.30
CFI^a^	0.930
TLI^b^	0.926
RMSEA^c^	0.055 (90% CI 0.047‐0.063)
SRMR^d^	0.103
GFI^e^	0.947

a CFI: comparative fit index.

b TLI: Tucker-Lewis index.

c RMSEA: root mean square error of approximation.

d SRMR: standardized root mean square residual.

e GFI: goodness-of-fit index.

The comparative fit index (CFI=0.930) and Tucker-Lewis index (TLI=0.926) both surpassed the benchmark of 0.90. The root mean square error of approximation (RMSEA) was 0.055 (90% CI 0.047‐0.063), indicating acceptable fit; however, the SRMR was 0.103, exceeding the conventional ≤0.08 cutoff and indicating that the model did not fully reproduce the observed item covariances. We did not apply post hoc modifications to avoid capitalizing on chance in a modest sample (N=196); the weak-loading items identified in Table 4 (AILkR, *λ*=0.22; AILeR, *λ*=0.27; AIEpR, *λ*=0.36) are likely contributors to the elevated SRMR and are flagged for revision in future validation work. The goodness-of-fit index (GFI=0.947) further supported the adequacy of the specified 5-factor structure for the Arabic NURS-DIAL.

**Table 4.** Standardized factor loadings and explained variance (*R*^2^) for Arabic Nursing Digital and AI Literacy Assessment (NURS-DIAL) items by subscale^a^.

Item code	Item label (short)	*λ*	*R* ^2^
Information Literacy (8 items)			
IL_a	Web search engines	0.63	0.397
IL_b	Academic databases	0.73	0.530
IL_c	Online collection tools	0.72	0.523
IL_d	Evaluate trustworthiness	0.90	0.819
IL_e	Organize information	0.79	0.618
IL_f	Academic integrity	0.76	0.574
IL_g	Share/acknowledge work	0.81	0.650
IL_h	Referencing	0.77	0.596
mHealth Literacy (6 items, reverse-coded)			
mHL_a_R	Compare health information	0.76	0.577
mHL_b_R	Awareness of health apps	0.90	0.807
mHL_c_R	Familiarity with health apps	0.89	0.789
mHL_d_R	Assess quality	0.85	0.723
mHL_e_R	Use for decisions	0.76	0.573
mHL_f_R	Access to support	0.46	0.212
AI Literacy (14 items)			
AIL_a	Distinguish smart devices	0.56	0.309
AIL_b_R	Don’t know if AI helps	0.42	0.179
AIL_d	Skillfully use AI	0.80	0.647
AIL_e_R	Hard to learn AI	0.27	0.074
AIL_f	Use AI for efficiency	0.75	0.558
AIL_h	Choose AI solutions	0.73	0.530
AIL_i	Choose best AI for task	0.86	0.748
AIL_j	Comply with ethics	0.90	0.816
AIL_k_R	Not alert to privacy	0.22	0.050
AIL_l	Alert to AI abuse	0.88	0.768
AIL_m	Ethical implications	0.87	0.750
AIL_n	Aware of AI biases	0.82	0.680
AIL_o	Protect personal data	0.66	0.438
AIL_p	Evaluate AI accuracy	0.71	0.499
Digital Wellbeing (5 items)			
DW_a	Comfortable with digital tech	0.84	0.698
DW_b	Recognize digital stress	0.71	0.509
DW_c	Online behavior consequences	0.83	0.687
DW_d	Against cyberbullying	0.84	0.705
DW_f	Access well-being services	0.73	0.531
AI Education Topics (19 items)			
AIE_a_R	Knowledge of AI apps	0.60	0.359
AIE_b_R	AI reduces errors	0.70	0.491
AIE_c_R	Training in AI ethics	0.64	0.411
AIE_d_R	AI disease risk	0.77	0.599
AIE_e_R	AI patient compliance	0.83	0.690
AIE_f_R	AI clinical decisions	0.63	0.402
AIE_g_R	AI in epidemics	0.75	0.569
AIE_h_R	AI predicts health	0.66	0.437
AIE_i_R	AI genetic risk	0.77	0.594
AIE_j_R	AI in emergency care	0.63	0.400
AIE_k_R	AI in research	0.69	0.475
AIE_l_R	AI preventive apps	0.74	0.542
AIE_m_R	Robotic surgery	0.63	0.399
AIE_n_R	AI and patient control	0.85	0.724
AIE_o_R	Chatbots	0.52	0.275
AIE_p_R	AI psychiatric diagnosis	0.36	0.127
AIE_q_R	Legal/regulatory issues	0.59	0.348
AIE_r_R	Data privacy	0.66	0.439
AIE_s_R	Critical evaluation	0.62	0.381

a Abbreviated item labels are presented to facilitate interpretation of standardized factor loadings and explained variance. Full item wording in Arabic, original English items, English backtranslations, response options, scoring directions, reverse-coded status, and item retention status are provided in Table S4 in Multimedia Appendix 1.

Table 4 presents the standardized factor loadings and explained variance (*R*^2^) for items across the five Arabic NURS-DIAL subscales. Most items showed moderate-to-high standardized factor loadings; however, several items had weaker loadings and should be evaluated in future validation studies.

The internal consistency reliability of the Arabic NURS-DIAL subscales is presented in Table 5. All domains showed good-to-excellent internal consistency. The Information Literacy subscale (8 items) had a Cronbach α of 0.890, the mHealth Literacy (6 items) and AI Literacy (14 items) subscales each showed good reliability (*α*=0.856), the Digital Wellbeing subscale (5 items) also had good reliability (*α*=0.868), and the AI Education Topics subscale (19 items) had excellent internal consistency (*α*=0.919). Coefficients were computed from all available responses; item-level valid sample sizes ranged from 149 to 156 for Information Literacy, 146 to 149 for mHealth Literacy, 131 to 144 for AI Literacy, 130 to 131 for Digital Wellbeing, and 111 to 129 for AI Education Topics (Tables S1 and S2 in Multimedia Appendix 1).

**Table 5.** Internal consistency reliability of the Arabic Nursing Digital and AI Literacy Assessment (NURS-DIAL) subscales^a^.

Subscale	Items	Valid n per item^b^	Cronbach α^c^	Interpretation
Information Literacy	8	149‐156	0.890	Good
mHealth Literacy	6	146‐149	0.856	Good
AI Literacy	14	131‐144	0.856	Good
Digital Wellbeing	5	130‐131	0.868	Good
AI Education Topics	19	111‐129	0.919	Excellent

a All *α* ≥0.70 indicate acceptable internal consistency.

b Valid n varied across items within each subscale due to missing data; the range of valid responses per item is reported.

c Cronbach α was computed using listwise deletion within each subscale.

Table 6 shows the intersubscale correlations of the Arabic NURS-DIAL and provides evidence of construct validity across the 5 domains. The information literacy subscale was positively correlated with mHealth literacy (*r*=0.263; *P*=.001), AI literacy (*r*=0.218; *P*=.009), and digital well-being (*r*=0.507; *P*<.001), but not correlated with AI education (*r*=−0.004). There were positive correlations between mHealth literacy and AI literacy (*r*=0.338; *P*<.001), digital well-being (*r*=0.244, *P*=.004), and AI education (*r*=0.290, *P*<.001). This indicates that higher mHealth literacy is somewhat associated with higher AI-related skills and education. AI literacy correlated significantly with digital well-being (*r*=0.402; *P*<.001) and AI education (*r*=0.327; *P*<.001). Digital well-being was positively related to AI Education (*r*=0.234; *P*<.01; small-to-moderate effect size).

**Table 6.** Inter-subscale correlations for the Arabic Nursing Digital and AI Literacy Assessment (NURS-DIAL; construct validity).

Subscale	Information Literacy	mHealth Literacy	AI Literacy	Digital Wellbeing	AI Education
Information Literacy	1.000	0.263^a^	0.218^a^	0.507^b^	−0.004
mHealth Literacy	0.263^a^	1.000	0.338^b^	0.244^a^	0.290^b^
AI Literacy	0.218^a^	0.338^b^	1.000	0.402^b^	0.327^b^
Digital Wellbeing	0.507^b^	0.244^a^	0.402^b^	1.000	0.234^a^
AI Education	−0.004	0.290^b^	0.327^b^	0.234^a^	1.000

a *P*<.01.

b *P*<.001 (2-tailed).

Table 7 presents the results of exploratory comparisons of Arabic NURS-DIAL subscale scores by sex and year of study. In Table 7, no statistically significant sex differences were found for information literacy, AI literacy, digital well-being, or AI education topics (all *P*>.05), with small-to-moderate effect sizes (absolute Cohen *d*=0.19-0.28). However, female students scored significantly higher than male students on mHealth literacy (female: mean 4.03, SD 0.64; male: mean 3.81, SD 0.64; *t*_142_=–2.02, Cohen *d*=–0.34; *P*=.045). Table 8, no significant differences were found across years of study for information literacy, mHealth literacy, AI literacy, or digital well-being (all *P>*.05); however, AI education topics differed significantly by year of study (*F*_₄₁₁₄_=5.91, *η*^2^=0.172; *P*<.001). These findings provide preliminary evidence of known-group validity and should be interpreted as exploratory.

**Table 7.** Exploratory comparisons of Arabic Nursing Digital and AI Literacy Assessment (NURS-DIAL) subscale scores in the current Saudi validation sample by sex of study^a^.

Subscale	Male, mean (SD)	Female, mean (SD)	*t* test (df)	*P* value	Cohen d
Information Literacy	3.00 (0.92)	2.83 (0.87)	1.20 (159)	.23	0.19
mHealth Literacy	3.81 (0.64)	4.03 (0.64)	−2.02 (142)	.045	−0.34
AI Literacy	5.44 (0.95)	5.67 (1.00)	−1.43 (141)	.15	−0.24
Digital Wellbeing	3.68 (0.69)	3.45 (1.02)	1.55 (134)	.13	0.27
AI Education Topics	3.62 (0.58)	3.80 (0.73)	−1.59 (128)	.11	−0.28

a Subscale scores are prorated means requiring ≥80% of items.

**Table 8.** Exploratory comparisons of Arabic Nursing Digital and AI Literacy Assessment (NURS-DIAL) subscale scores in the current Saudi validation sample by year of study^a^.

Subscale	*F* test (df)	*P* value	*η* ^2^
Information Literacy	1.34 (4, 141)	.26	0.037
mHealth Literacy	0.81 (4, 127)	.52	0.025
AI Literacy	1.04 (4, 126)	.39	0.032
Digital Wellbeing	1.10 (4, 120)	.36	0.035
AI Education Topics	5.91 (4, 114)	<.001	0.172

a Subscale scores are prorated means requiring ≥80% of items.

Table 9 summarizes the internal consistency reliability, standardized factor-loading ranges, and global CFA fit indices for the Arabic NURS-DIAL. All 5 subscales demonstrated good-to-excellent internal consistency. Most items showed moderate-to-high factor loadings; however, several items had weaker loadings. The overall 5-factor model showed acceptable fit on most indices, although the SRMR exceeded the conventional cutoff.

**Table 9.** Summary of the internal consistency reliability, standardized factor-loading ranges, and global confirmatory factor analysis (CFA) fit indices for the Arabic NURS-DIAL^a^.

Subscale	Final items	Cronbach α	Standardized loading range
Information Literacy	8	0.890	0.63‐0.90
mHealth Literacy	6	0.856	0.46‐0.90
AI Literacy	14	0.856	0.22‐0.90
Digital Wellbeing	5	0.868	0.71‐0.84
AI Education Topics	19	0.919	0.36‐0.85

a The global fit indices for the 52-item, 5-factor model were as follows: comparative fit index was 0.930, Tucker-Lewis index was 0.926, root mean square error of approximation was 0.055 (90% CI 0.047-0.063), standardized root mean square residual was 0.103, and goodness-of-fit index was 0.947. Detailed comparative fit index results, item-level loadings, and reliability results are shown in Tables 3-5.

## Discussion

### Principal Findings

This study provides preliminary evidence supporting the Arabic NURS-DIAL as a culturally adapted instrument for assessing digital and AI literacy among nursing students in Saudi Arabia. The findings support content validity; internal consistency reliability; and aspects of structural, construct, and known-groups validity. The implications of the findings should be interpreted considering the study’s methodological limitations.

### Cross-Cultural Adaptation of the Arabic NURS-DIAL

The Arabic NURS-DIAL was developed through a structured translation and cultural adaptation process, including forward translation, reconciliation, back-translation, expert review, content validation, and student pretesting, in accordance with Beaton et al [15]. The content-validity findings and student feedback support the clarity and cultural acceptability of the Arabic version in this sample. The Arabic and English versions of the questionnaire, English backtranslations, response options, scoring directions, and item-retention status are provided in Table S4 in Multimedia Appendix 1.

The translation and adaptation process is important when assessing digital and AI literacy in Arabic-speaking nursing students. Previous studies in the Middle East and North Africa have explored attitudes toward AI, readiness to use AI, AI literacy, and willingness to use AI. Many of these studies used tools translated from English into Arabic but did not clearly explain how the Arabic versions were culturally adapted or psychometrically tested [9,19]. This study offers preliminary support for an Arabic tool that covers information literacy, mHealth literacy, AI literacy, digital well-being, and perceived AI education needs. Further studies are needed with larger and more diverse groups of Arabic-speaking nursing students.

### Structural Validity

The CFA showed initial support for the 5-factor model. Most fit indices were acceptable, but the SRMR was above the recommended cutoff. The 3 retained items AILeR, AILkR, and AIEpR had low loadings. This was related to the higher SRMR. These items need further review in a larger independent sample. No post hoc changes were made because the sample size was limited.

### Reliability and Construct Validity

The subscales showed good reliability. Information literacy, mHealth literacy, AI literacy, and digital well-being had good internal consistency, while AI education topics had excellent internal consistency. This means that the items within each section were generally consistent in measuring the same area. These results support the use of the Arabic NURS-DIAL with nursing students for research and low-stakes educational assessment [20,21].

Construct validity was also supported by the relationships between the subscales. Information literacy, mHealth literacy, AI literacy, and digital well-being were positively related, suggesting that students who were more confident in 1 area often reported stronger skills in other digital areas. However, AI education topics showed weaker relationships with the other areas, especially information literacy. This suggests that students’ views about what AI topics should be included in nursing education are related to, but different from, their digital and AI skills. Overall, the findings suggest that the NURS-DIAL measures are related but separate areas of digital and AI literacy [22,23].

### Known-Group Validity

The known-groups analysis provided early, partial support for the validity of the Arabic NURS-DIAL. Female students scored higher than male students in mHealth literacy, which may suggest that the scale can identify sex-related differences in the use of digital health tools. This finding is consistent with Lupton and Maslen [24], who reported that women often use digital technologies to seek health information and support. However, because this difference was small, it should be interpreted cautiously. No clear sex differences were found in information literacy, AI literacy, digital well-being, or AI education topics. This is in line with Alshowkan and Shdaifat [13], who suggested that sex differences in digital and AI literacy may be decreasing as students have more similar exposure to technology in education.

Students’ views about AI education topics differed by year of study. This may indicate that students at different stages of the nursing program consider different AI topics important to their education. Bakarman et al [12] suggested that students may develop a greater understanding of and openness to AI as they gain clinical experience and progress through nursing education. However, the cross-sectional design did not allow the assessment of changes in students’ views over time.

The findings of El Arab et al [9] similarly showed that nursing students and nurses in the Middle East and North Africa generally had positive attitudes toward AI but reported gaps in knowledge, readiness, and experience using AI in clinical practice. In the present study, the Arabic NURS-DIAL identified lower AI literacy in areas related to privacy and the perceived difficulty of learning AI.

Apart from differences in AI education topics by year of study, no other year-group differences were observed. These exploratory findings require replication in larger and more diverse samples.

### Implications for Nursing Curricula and AI-Enabled Education

The findings suggest that the Arabic NURS-DIAL may help educators identify areas in which nursing students require further support in digital and AI literacy.

First, the 5 subscales may provide information for educators and curriculum committees about students’ strengths and learning needs. In this study, students had lower scores in information literacy, suggesting a need for learning activities focused on searching for information, evaluating information sources, and referencing academic work. Students also showed lower AI literacy in areas related to AI privacy and the perceived difficulty of learning AI. Educational activities addressing the safe use of AI, privacy, and the critical evaluation of AI-generated information may therefore be beneficial.

Second, students in different years of the nursing program had different views about which AI topics were important. AI content can be introduced gradually as students move through the program. Topics such as clinical decision support, ethics, and checking AI-generated information are more suitable for students with more clinical experience. Assessing students at different stages can also help faculty see whether curriculum changes improve digital and AI literacy over time.

There are currently no normative data or validated cutoff scores for the NURS-DIAL. Therefore, scores should be used to identify learning needs and support teaching, rather than for diagnosis, institutional comparison, or high-stakes decisions.

### Strengths and Limitations

This study has several strengths. The Arabic NURS-DIAL was translated and culturally adapted using an established framework, reviewed by a multidisciplinary expert panel, and pretested with nursing students. The study also evaluated several psychometric properties, including content validity, structural validity, internal consistency reliability, construct validity, and known-groups validity. In addition, CFA was conducted using an estimator appropriate for ordinal data.

The findings should be interpreted in light of several limitations. The sample was recruited by convenience sampling from 1 university and consisted of undergraduate students in general nursing, which limits generalizability to other institutions, educational levels, specialties, and Arabic-speaking populations. The sample size was also limited in relation to the 52-item, 5-factor CFA model. Therefore, the factor structure should be considered preliminary. Test-retest reliability, criterion-related validity against objective digital or AI competence measures, and measurement invariance across groups were not assessed. Future studies should use multi-institutional and more diverse samples to confirm the factor structure, examine measurement performance across subgroups, and evaluate stability over time.

### Conclusions

The findings provide preliminary support for the Arabic NURS-DIAL as a measure of digital and AI literacy among nursing students in the Saudi setting studied. The evidence for content validity, structural validity, internal consistency reliability, and construct validity was obtained. The limited sample size and single-institution design require cautious interpretation. At present, the instrument is most appropriate for exploratory research and formative assessment in comparable settings. Multi-institutional studies are needed to confirm the factor structure and establish its suitability for broader use.

## Supplementary material

10.2196/106044 Multimedia Appendix 1 Supplementary tables: Table S1 (item-level missing-data frequencies and percentages by subscale), Table S2 (missingness pattern by questionnaire position), Table S3 (sensitivity analyses comparing primary confirmatory factor analysis results with complete-case and multiple-imputation results), and Table S4 (Arabic Nursing Digital and AI Literacy Assessment [NURS-DIAL] questionnaire with full item wording, English backtranslations, and removal rationale for dropped items).

## Abbreviations

CFA: confirmatory factor analysis
CFI: comparative fit index
GCC: Gulf Cooperation Council
GFI: goodness-of-fit index
I-CVI: item-level content validity index
LMS: learning management system
mHealth: mobile health
NURS-DIAL: Nursing Digital and AI Literacy Assessment
RMSEA: root mean square error of approximation
SRMR: standardized root mean square residual
TLI: Tucker-Lewis index
WLSMV: weighted least squares mean and variance
