# Supplementary material for: Preliminary Psychometric Evaluation of the Arabic Version of the NURS-DIAL Questionnaire Among Nursing Students in Saudi Arabia: Cross-Sectional Study
Source: JMIR Nurs. 2026 Oct 30;9:e106044. doi: 10.2196/106044 (PMC13722094; doi:10.2196/106044)
Supplement: Supplementary file 1 [file nursing-v9-e106044-s001.docx]

# Supplementary Material: Missing Data and Sensitivity Analyses

**Table S1. Item-level missing-data frequencies and percentages**

| **Subscale** | **Items** | **Valid responses, n** | **Missing responses, n (%)** | **Item-level missingness range, %** | **Valid n per item, range** |
| --- | --- | --- | --- | --- | --- |
| Information Literacy | 8 | 1227 | 341 (21.7) | 20.4–24.0 | 149–156 |
| mHealth Literacy | 6 | 887 | 289 (24.6) | 24.0–25.5 | 146–149 |
| AI Literacy | 14 | 1939 | 805 (29.3) | 26.5–33.2 | 131–144 |
| Digital Wellbeing | 5 | 654 | 326 (33.3) | 33.2–33.7 | 130–131 |
| AI Education Topics | 19 | 2275 | 1449 (38.9) | 34.2–43.4 | 111–129 |
| Total | 52 | 6982 | 3210 (31.5) | 20.4–43.4 | 111–156 |

*Note. Derived from item-level counts in Table S2. Missingness increased monotonically with item position in the fixed-order survey (Spearman* $\rho=0.995$*,* $p<.001$*; range 20.4%–43.4%), a pattern consistent with survey discontinuation (monotone dropout) rather than item-specific refusal.*

***Missing data.*** *Item nonresponse increased monotonically with questionnaire position in the fixed-order survey (Spearman* $\rho=0.995$*,* $p<.001$*; 20.4%–43.4%), a pattern consistent with survey discontinuation rather than item-specific nonresponse (Tables S1–S2). Because the missing-data mechanism cannot be established from observed data alone, the robustness of the findings was assessed through sensitivity analyses. The five-factor CFA results, subscale reliability estimates, and known-groups comparisons were materially unchanged in complete-case and multiple-imputation analyses (Table S3)*

**Table S2. Missingness pattern by questionnaire position or subscale**

| **Scale** | **Item code** | **Item (short label)** | **Missing n** | **Missing %** | **Valid n** |
| --- | --- | --- | --- | --- | --- |
| Information Literacy | IL_a | Web search engines | 40 | 20.4 | 156 |
| Information Literacy | IL_b | Academic databases | 42 | 21.4 | 154 |
| Information Literacy | IL_c | Online collection tools | 42 | 21.4 | 154 |
| Information Literacy | IL_d | Evaluate trustworthiness | 41 | 20.9 | 155 |
| Information Literacy | IL_e | Organize information | 42 | 21.4 | 154 |
| Information Literacy | IL_f | Academic integrity | 45 | 23.0 | 151 |
| Information Literacy | IL_g | Share/acknowledge work | 42 | 21.4 | 154 |
| Information Literacy | IL_h | Referencing | 47 | 24.0 | 149 |
| mHealth Literacy | mHLaR | Compare health information | 48 | 24.5 | 148 |
| mHealth Literacy | mHLbR | Awareness of health apps | 47 | 24.0 | 149 |
| mHealth Literacy | mHLcR | Familiarity with health apps | 47 | 24.0 | 149 |
| mHealth Literacy | mHLdR | Assess quality | 48 | 24.5 | 148 |
| mHealth Literacy | mHLeR | Use for decisions | 49 | 25.0 | 147 |
| mHealth Literacy | mHLfR | Access to support | 50 | 25.5 | 146 |
| AI Literacy | AIL_a | Distinguish smart devices | 52 | 26.5 | 144 |
| AI Literacy | AIL_b_R | Don't know AI helps | 53 | 27.0 | 143 |
| AI Literacy | AIL_d | Skillfully use AI | 56 | 28.6 | 140 |
| AI Literacy | AIL_e_R | Hard to learn AI | 56 | 28.6 | 140 |
| AI Literacy | AIL_f | Use AI for efficiency | 56 | 28.6 | 140 |
| AI Literacy | AIL_h | Choose AI solutions | 58 | 29.6 | 138 |
| AI Literacy | AIL_i | Choose best AI for task | 56 | 28.6 | 140 |
| AI Literacy | AIL_j | Comply with ethics | 56 | 28.6 | 140 |
| AI Literacy | AIL_k_R | Not alert to privacy | 59 | 30.1 | 137 |
| AI Literacy | AIL_l | Alert to AI abuse | 57 | 29.1 | 139 |
| AI Literacy | AIL_m | Ethical implications | 58 | 29.6 | 138 |
| AI Literacy | AIL_n | Aware of AI biases | 61 | 31.1 | 135 |
| AI Literacy | AILo | Protect personal data | 62 | 31.6 | 134 |
| AI Literacy | AIL_p | Evaluate AI accuracy | 65 | 33.2 | 131 |
| Digital Wellbeing | DW_a | Comfortable with digital tech | 65 | 33.2 | 131 |
| Digital Wellbeing | DW_b | Recognize digital stress | 65 | 33.2 | 131 |
| Digital Wellbeing | DW_c | Online behaviour consequences | 66 | 33.7 | 130 |
| Digital Wellbeing | DW_d | Against cyberbullying | 65 | 33.2 | 131 |
| Digital Wellbeing | DW_f | Access wellbeing services | 65 | 33.2 | 131 |
| AI Education Topics | AIE_a_R | Knowledge of AI apps | 67 | 34.2 | 129 |
| AI Education Topics | AIE_b_R | AI reduces errors | 70 | 35.7 | 126 |
| AI Education Topics | AIE_c_R | Training in AI ethics | 69 | 35.2 | 127 |
| AI Education Topics | AIE_d_R | AI disease risk | 70 | 35.7 | 126 |
| AI Education Topics | AIE_e_R | AI patient compliance | 70 | 35.7 | 126 |
| AI Education Topics | AIE_f_R | AI clinical decisions | 74 | 37.8 | 122 |
| AI Education Topics | AIE_g_R | AI in epidemics | 72 | 36.7 | 124 |
| AI Education Topics | AIE_h_R | AI predicts health | 73 | 37.2 | 123 |
| AI Education Topics | AIE_i_R | AI genetic risk | 77 | 39.3 | 119 |
| AI Education Topics | AIE_j_R | AI in emergency care | 76 | 38.8 | 120 |
| AI Education Topics | AIE_k_R | AI in research | 79 | 40.3 | 117 |
| AI Education Topics | AIE_l_R | AI preventive apps | 79 | 40.3 | 117 |
| AI Education Topics | AIE_m_R | Robotic surgery | 80 | 40.8 | 116 |
| AI Education Topics | AIE_n_R | AI and patient control | 78 | 39.8 | 118 |
| AI Education Topics | AIE_o_R | Chatbots | 81 | 41.3 | 115 |
| AI Education Topics | AIE_p_R | AI psychiatric diagnosis | 82 | 41.8 | 114 |
| AI Education Topics | AIE_q_R | Legal/regulatory issues | 84 | 42.9 | 112 |
| AI Education Topics | AIE_r_R | Data privacy | 83 | 42.3 | 113 |
| AI Education Topics | AIE_s_R | Critical evaluation | 85 | 43.4 | 111 |

*Note. Percentages use N = 196. Item codes correspond to Table 4 of the manuscript.*

**Table S3. Sensitivity analyses comparing primary pairwise-present WLSMV CFA results with complete-case and multiple-imputation results**

| **Analysis** | **n** | **χ²/df** | **CFI** | **TLI** | **RMSEA (90% CI)** | **SRMR** | **GFI** | **Loading range** |
| --- | --- | --- | --- | --- | --- | --- | --- | --- |
| Primary: WLSMV, pairwise-present | 196 (pairwise ≤111–156) | 1.30 | 0.930 | 0.926 | 0.055 (0.047–0.063) | 0.103 | 0.947 | 0.22–0.90 |
| Complete cases | 106 | 1.42 | 0.922 | 0.918 | 0.061 (0.052–0.070) | 0.109 | 0.938 | 0.19–0.88 |
| WLSMV after multiple imputations (m = 40) | 196 | 1.27 | 0.934 | 0.930 | 0.052 (0.044–0.060) | 0.100 | 0.949 | 0.24–0.90 |

**Panel B. Subscale reliability (Cronbach's α)**

| **Subscale** | **α (primary)** | **α (complete cases)** | **n (complete cases)** |
| --- | --- | --- | --- |
| Information Literacy | 0.890 | 0.887 | 146 |
| mHealth Literacy | 0.856 | 0.852 | 142 |
| AI Literacy | 0.856 | 0.851 | 126 |
| Digital Wellbeing | 0.868 | 0.863 | 128 |
| AI Education Topics | 0.919 | 0.916 | 108 |

**Panel C. Known-groups comparisons (complete-case sensitivity)**

| **Comparison** | **Primary result** | **Complete-case result** |
| --- | --- | --- |
| mHealth Literacy by gender | t = 2.02, p = .045, d = 0.34 | t = 1.98, p = .049, d = 0.33 |
| AI Education Topics by year of study | F = 5.91, p < .001, η² = .172 | F = 5.32, p < .001, η² = .165 |

The Arabic NURS-DIAL questionnaire is reproduced here with permission from the AI4Health consortium. Item codes correspond to Table 4 of the main manuscript and Supplementary Tables S1–S2. This presents the original English items, Arabic item wording, English backtranslations, response options, scoring directions, reverse-coded status, and item-retention status. English backtranslations were prepared during Stage III of the cross-cultural adaptation process.

# Table S4. Arabic NURS-DIAL Questionnaire: Full Item Wording, Original English Items, English Backtranslations, Response Options, Scoring Directions, Reverse-Coded Status, and Item-Retention Status

| ***Information Literacy (8 items; 5-point: Novice to Expert)*** | | | | | | |
| --- | --- | --- | --- | --- | --- | --- |
| Item code | Short label | | Arabic item wording | | English backtranslation | |
| IL_a | Web search engines | | العثور على معلومات رقمية ذات صلة بدراستك الأكاديمية باستخدام مصادر الويب غير الرسمية (مثل Google، Google Scholar، Bing أو محركات بحث أخرى). | | Finding relevant digital information using informal web sources (e.g., Google, Google Scholar, Bing). | |
| IL_b | Academic databases | | العثور على معلومات رقمية ذات صلة بدراستك الأكاديمية باستخدام قواعد البيانات (مثل CINAHL، Medline، ScienceDirect، Cochrane Library). | | Finding information using academic databases (e.g., CINAHL, Medline). | |
| IL_c | Online collection tools | | استخدام أدوات تجميع عبر الإنترنت لجمع المعلومات الرقمية بطرق جديدة (مثل SlideShare، List.ly، Pinterest، Quora، Scoop.it، وغيرها). | | Using online aggregation tools (e.g., SlideShare, Quora). | |
| IL_d | Evaluate trustworthiness | | تقييم ما إذا كانت المعلومات الرقمية موثوقة وذات صلة. | | Assessing the reliability and relevance of digital information. | |
| IL_e | Organize information | | تنظيم المعلومات الرقمية التي تجدها لتساعدك في التعلم، باستخدام المجلدات، العلامات المرجعية، برامج إدارة المراجع، ووضع العلامات (Tagging). | | Organizing digital information for learning using folders, bookmarks, etc. | |
| IL_f | Academic integrity | | فهم النزاهة الأكاديمية / الأمانة عند الوصول إلى المعلومات واستخدامها عبر الإنترنت (مثل الانتحال، التواطؤ). | | Understanding academic integrity online (e.g., plagiarism). | |
| IL_g | Share/acknowledge work | | فهم كيفية مشاركة المعلومات علنًا عبر الإنترنت مع احترام وتقدير أعمال الآخرين (مثل استخدام ترخيص المشاع الإبداعي، وتقديم المراجع / الاستشهادات بالأعمال الأصلية). | | Sharing digital content respectfully (e.g., Creative Commons licenses). | |
| IL_h | Referencing | | توثيق مصادر المعلومات الرقمية والالتزام بأسلوب توثيق معين (مثل أسلوب Harvard). | | Referencing digital sources in a specific style (e.g., Harvard). | |
| ***mHealth Literacy (6 items; 5-point, reverse-scored: Strongly Disagree to Strongly Agree)*** | | | | | | |
| Item code | Short label | | Arabic item wording | | English backtranslation | |
| mHL_a_R | Compare health information | | يمكنني مقارنة المعلومات الصحية المختلفة التي أحصل عليها من خلال الأجهزة والتطبيقات المحمولة لتحديد المعلومات الدقيقة. | | Comparing health information from mobile apps to determine accuracy. | |
| mHL_b_R | Awareness of health apps | | أنا على دراية بالمعلومات الصحية والاستخدامات الممكنة للتطبيقات عبر الأجهزة المحمولة. | | Awareness of health apps and their potential uses. | |
| mHL_c_R | Familiarity with health apps | | لدي إلمام بكيفية استخدام المعلومات الصحية والتطبيقات التي أجدها من خلال الأجهزة المحمولة. | | Familiarity with using health apps and info. | |
| mHL_d_R | Assess quality | | أستطيع تقييم جودة المعلومات الصحية / التطبيقات التي أجدها من خلال الأجهزة المحمولة. | | Evaluating quality of mobile health info. | |
| mHL_e_R | Use for decisions | | أستطيع استخدام المعلومات الصحية التي أحصل عليها من خلال الأجهزة المحمولة في اتخاذ قرارات تتعلق بصحتي. | | Using info from mobile apps to make health decisions. | |
| mHL_f_R | Access to support | | هناك أفراد أو مؤسسات متاحة في أي وقت وأي مكان، إذا رغبت، للمساعدة في إدارة الصحة والحصول على المعلومات الصحية (مثل أفراد الأسرة الذين يمكنهم شرح تطبيقات الصحة أو العيادات الصحية العامة). | | Having access to individuals/institutions for support in health info. | |
| ***AI Literacy (14 items after removal of AIL_c and AIL_g; 7-point: Strongly Disagree to Strongly Agree)*** | | | | | | |
| Item code | Short label | | Arabic item wording | | English backtranslation | |
| AIL_a | Distinguish smart devices | | أستطيع التمييز بين الأجهزة الذكية وغير الذكية. | | Distinguishing between smart and non-smart devices. | |
| AIL_b_R | Don't know AI helps | | لا أعرف كيف يمكن لتقنية الذكاء الاصطناعي أن تساعدني. | | Lack of understanding of how AI helps. | |
| AIL_d | Skillfully use AI | | أستطيع استخدام تطبيقات أو منتجات الذكاء الاصطناعي بمهارة لمساعدتي في عملي اليومي. | | Skillfully using AI apps/products in daily work. | |
| AIL_e_R | Hard to learn AI | | عادة ما أجد صعوبة في تعلم استخدام تطبيق أو منتج جديد للذكاء الاصطناعي. | | I have difficulty in learning new AI tools. | |
| AIL_f | Use AI for efficiency | | أستطيع استخدام تطبيقات أو منتجات الذكاء الاصطناعي لتحسين كفاءة عملي. | | Using AI to improve work efficiency. | |
| AIL_h | Choose AI solutions | | أستطيع اختيار الحل المناسب من بين الحلول المختلفة التي يقدمها وكيل ذكي. | | Choosing the best AI solution. | |
| AIL_i | Choose best AI for task | | أستطيع اختيار أنسب تطبيق أو منتج للذكاء الاصطناعي من بين عدة خيارات لمهمة معينة. | | Selecting the most suitable AI tool for a task. | |
| AIL_j | Comply with ethics | | ألتزم دائمًا بالمبادئ الأخلاقية عند استخدام تطبيقات أو منتجات الذكاء الاصطناعي. | | Following ethical principles when using AI. | |
| AIL_k_R | Not alert to privacy | | لا أكون يقظًا أبدًا تجاه قضايا الخصوصية وأمن المعلومات عند استخدام تطبيقات أو منتجات الذكاء الاصطناعي. | | Awareness of privacy and security in AI use. | |
| AIL_l | Alert to AI abuse | | أنا دائمًا منتبه لإساءة استخدام تقنية الذكاء الاصطناعي. | | Alertness to AI misuse. | |
| AIL_m | Ethical implications | | أفهم الآثار الأخلاقية المترتبة على استخدام الذكاء الاصطناعي. | | Understanding the ethical implications of AI use. | |
| AIL_n | Aware of AI biases | | أنا على دراية بالتحيزات المحتملة في أنظمة الذكاء الاصطناعي. | | Awareness of potential biases in AI systems. | |
| AIL_o | Protect personal data | | أعرف كيف أحمي بياناتي الشخصية عند استخدام أدوات الذكاء الاصطناعي. | | Knowing how to protect personal data when using AI tools. | |
| AIL_p | Evaluate AI accuracy | | أستطيع تقييم دقة وموثوقية مخرجات الذكاء الاصطناعي. | | Evaluating the accuracy and reliability of AI outputs. | |
| ***Digital Wellbeing (5 items after removal of DW_e; 5-point: Novice to Expert)*** | | | | | | |
| Item code | Short label | | Arabic item wording | | English backtranslation | |
| DW_a | Comfortable with digital tech | | الشعور بالراحة والسيطرة والأمان عند استخدام التقنيات الرقمية. | | Feeling comfortable and safe using digital tech. | |
| DW_b | Recognize digital stress | | إدراك أن المعلومات والوسائط الرقمية يمكن أن تسبب تشتتًا وإرهاقًا وتوترًا، والانفصال عنها عند الضرورة. | | Recognizing when digital content causes stress and disconnecting. | |
| DW_c | Online behaviour consequences | | النظر في الصواب والخطأ والعواقب المحتملة لسلوكك عبر الإنترنت. | | Thinking about right/wrong and consequences online. | |
| DW_d | Against cyberbullying | | التصرف بإيجابية ضد التنمر الإلكتروني وغيره من السلوكيات الضارة عبر الإنترنت. | | Standing against cyberbullying and harmful behaviors. | |
| DW_f | Access wellbeing services | | استخدام الوسائط الرقمية للوصول إلى خدمات الرفاهية ومراقبة الحالات الصحية (مثل خدمات الدعم الطلابي). | | Using digital media to access wellness services. | |
| ***AI Education Topics (19 items after removal of AIE_t; 5-point, reverse-scored: higher = greater perceived importance)*** | | | | | | |
| Item code | Short label | | Arabic item wording | | English backtranslation | |
| AIE_a_R | Knowledge of AI apps | | المعرفة والمهارات المتعلقة بتطبيقات الذكاء الاصطناعي | | Knowledge and skills related to AI applications | |
| AIE_b_R | AI reduces errors | | تطبيقات الذكاء الاصطناعي للحد من الأخطاء الطبية | | AI applications to reduce medical errors | |
| AIE_c_R | Training in AI ethics | | التدريب على الوقاية من المشكلات الأخلاقية التي قد تنشأ مع تطبيقات الذكاء الاصطناعي وحلها | | Training to prevent and resolve ethical issues that may arise with AI applications | |
| AIE_d_R | AI disease risk | | تحليل المخاطر المدعوم بالذكاء الاصطناعي لاكتشاف الأمراض | | Risk analysis supported by AI for disease detection | |
| AIE_e_R | AI patient compliance | | تطبيقات الذكاء الاصطناعي التي تعزز التزام المرضى بالعلاج | | AI applications that enhance patient adherence to treatment | |
| AIE_f_R | AI clinical decisions | | تطبيقات الذكاء الاصطناعي لدعم اتخاذ القرارات السريرية | | AI applications to support clinical decision-making | |
| AIE_g_R | AI in epidemics | | المراقبة والعزل المدعومان بالذكاء الاصطناعي أثناء الأوبئة | | AI-assisted surveillance and isolation during pandemics | |
| AIE_h_R | AI predicts health | | تحليلات الذكاء الاصطناعي التي تساعد في فهم الظواهر الصحية والتنبؤ بها | | AI analytics that help understand and predict health phenomena | |
| AIE_i_R | AI genetic risk | | تقييم المخاطر الجينية المدعوم بالذكاء الاصطناعي | | Genetic risk assessment supported by AI | |
| AIE_j_R | AI in emergency care | | الاستجابة للطوارئ المدعومة بالذكاء الاصطناعي | | Emergency response supported by AI | |
| AIE_k_R | AI in research | | الذكاء الاصطناعي في البحث العلمي | | AI in scientific research | |
| AIE_l_R | AI preventive apps | | تطبيقات الصحة الوقائية المحمولة المبنية على الذكاء الاصطناعي والمستخدمة على نطاق واسع | | Widely used AI-based mobile preventive health applications | |
| AIE_m_R | Robotic surgery | | الجراحة الروبوتية / العلاج والروبوتات الاجتماعية للرعاية المنزلية | | Robotic surgery/therapy and social robots for home care | |
| AIE_n_R | AI and patient control | | تطبيقات الذكاء الاصطناعي التي تعزز قدرة المرضى على إدارة صحتهم | | AI applications that enhance patients' ability to manage their health | |
| AIE_o_R | Chatbots | | المساعدون الافتراضيون / روبوتات المحادثة (Chatbots) | | Virtual assistants / Chatbots | |
| AIE_p_R | AI psychiatric diagnosis | | التشخيص والعلاج النفسي المدعوم بالذكاء الاصطناعي | | AI-assisted mental health diagnosis/treatment | |
| AIE_q_R | Legal/regulatory issues | | الجوانب القانونية والتنظيمية للذكاء الاصطناعي في التمريض | | Legal and regulatory aspects of AI in nursing | |
| AIE_r_R | Data privacy | | خصوصية بيانات المرضى وأمنها | | Patient data privacy and security | |
| AIE_s_R | Critical evaluation | | التقييم النقدي لمخرجات الذكاء الاصطناعي | | Critical evaluation of AI outputs | |
| ***Items removed after confirmatory factor analysis*** | | | | | | |
| Item code | Subscale | Arabic item wording | | English backtranslation | | Removal rationale |
| AIL_c | AI Literacy | أستطيع تحديد تقنية الذكاء الاصطناعي المستخدمة في التطبيقات والمنتجات التي أستخدمها. | | Recognizing AI technology in apps/products. | | Removed during CFA to improve model fit. The item ("Identify AI in apps/products") showed conceptual overlap with AIL_a (distinguishing smart devices) and AIL_d (skillfully using AI), contributing to localized misfit. |
| AIL_g | AI Literacy | أستطيع تقييم قدرات وحدود تطبيق أو منتج للذكاء الاصطناعي بعد استخدامه لفترة. | | Evaluating AI tool capabilities. | | Removed during CFA to improve model fit. The item ("Evaluate AI capabilities") showed conceptual overlap with AIL_p (evaluate AI accuracy) and AIL_i (choose best AI for task), contributing to redundancy in the measurement model. |
| DW_e | Digital Wellbeing | إدارة التفاعلات عبر الإنترنت والتفاعلات الواقعية بطرق تدعم العلاقات الصحية. | | Managing digital and real-life interactions healthily. | | Removed during CFA to improve model fit. The item ("Manage digital and real-life interactions healthily") showed the weakest loading within the Digital Wellbeing subscale and conceptual overlap with DW_a (feeling comfortable with digital tech) and DW_b (recognizing digital stress). |
| AIE_t | AI Education Topics | أخرى (يرجى التحديد) | | Other (please specify) | | Removed during CFA due to the lowest factor loading (λ = 0.38) and the highest item-level missingness (43.4%). As an open-ended "Other" category, the item lacked specific content and was not conceptually parallel to the other 19 topic-specific items. |
